# Near-universal hospitalization of US emergency department patients with cancer and febrile neutropenia

**DOI:** 10.1371/journal.pone.0216835

**Published:** 2019-05-23

**Authors:** Christopher W. Baugh, Mohammad Kamal Faridi, Emily L. Mueller, Carlos A. Camargo, Daniel J. Pallin

**Affiliations:** 1 Department of Emergency Medicine, Brigham and Women’s Hospital, Boston, Massachusetts, United States of America; 2 Harvard Medical School, Boston, Massachusetts, United States of America; 3 Department of Emergency Medicine, Massachusetts General Hospital, Boston, Massachusetts, United States of America; 4 Department of Pediatrics, Indiana University School of Medicine, Indianapolis, Indiana, United States of America; University of Bern, SWITZERLAND

## Abstract

**Importance:**

Febrile neutropenia (FN) is the most common oncologic emergency and is among the most deadly. Guidelines recommend risk stratification and outpatient management of both pediatric and adult FN patients deemed to be at low risk of complications or mortality, but our prior single-center research demonstrated that the vast majority (95%) are hospitalized.

**Objective:**

From a nationwide perspective, to determine the proportion of cancer patients of all ages hospitalized after an emergency department (ED) visit for FN, and to analyze variability in hospitalization rates. Our *a priori* hypothesis was that >90% of US cancer-associated ED FN visits would end in hospitalization.

**Design:**

Analysis of data from the Nationwide Emergency Department Sample, 2006–2014.

**Setting:**

Stratified probability sample of all US ED visits.

**Participants:**

Inclusion criteria were: (1) Clinical Classification Software code indicating cancer, (2) diagnostic code indicating fever, and (3) diagnostic code indicating neutropenia. We excluded visits ending in transfer.

**Exposure:**

The hospital at which the visit took place.

**Main outcomes and measures:**

Our main outcome is the proportion of ED FN visits ending in hospitalization, with an *a priori* hypothesis of >90%. Our secondary outcomes are: (a) hospitalization rates among subsets, and (b) proportion of variability in the hospitalization rate attributable to which hospital the patient visited, as measured by the intra-class correlation coefficient (ICC).

**Results:**

Of 348,868 visits selected to be representative of all US ED visits, 94% ended in hospitalization (95% Confidence Interval [CI] 93–94%). Each additional decade of age conferred 1.23x increased odds of hospitalization. Those with private (92%), self-pay (92%), and other (93%) insurance were less likely to be hospitalized than those with public insurance (95%, odds ratios [OR] 0.74–0.76). Hospitalization was least likely at non-metropolitan hospitals (84%, OR 0.15 relative to metropolitan teaching hospitals), and was also less likely at metropolitan non-teaching hospitals (94%, OR 0.64 relative to metropolitan teaching hospitals). The ICC adjusted for hospital random effects and patient and hospital characteristics was 26% (95%CI 23–29%), indicating that 26% of the variability in hospitalization rate was attributable to which hospital the patient visited.

**Conclusions and relevance:**

Nearly all cancer-associated ED FN visits in the US end in hospitalization. Inter-hospital variation in hospitalization practices explains 26% of the limited variability in hospitalization decisions. Simple, objective tools are needed to improve risk stratification for ED FN patients.

## Introduction

The National Cancer Institute has made research on oncologic emergencies a priority.[1,2] Large-scale, multi-state research on emergency department (ED) use by adults and children with cancer was limited until the recent publication of findings from the Nationwide Emergency Department Sample (NEDS).[3,4] Though cancer chemotherapy-associated febrile neutropenia (FN) is the most common oncologic emergency, one of these publications (the one focused on adults) did not address FN.[4] This likely resulted from the lack of a unique code for FN in the *International Classification of Diseases* (ICD9).

FN can be deadly, but many patients are stable and guidelines recommend discharge with oral antibiotics in adults deemed to be at low risk by a Multinational Association for Supportive Care in Cancer (MASCC) score >20, and absence of other indicators of risk.[5,6] At our cancer-center-associated ED, 25% of adults with FN were classified as low risk by MASCC,[7] and in the original MASCC study, 63% were.[8] Furthermore, pediatric-specific guidelines also recommend risk stratification and outpatient management of a low-risk cohort.[9] Despite this, we found that only 5% of all adult FN patients are discharged to home from our ED.[7,10] This discordance of guidelines and practice is likely multifactorial, in part due to lack of guideline awareness, the complexity of the guidelines, and because the MASCC score has a negative predictive value for complications of only 83% and therefore is not trusted.[11,12] However, we suspected that differences in regional practice patterns, hospital teaching status, case mix or cancer staging could possibly impact hospitalization rates for low-risk cancer patients with FN.

We analyzed NEDS data in order to describe hospitalization practices for ED patients with cancer-associated FN on a nationwide scale. Based on our previous work, we hypothesized that >90% of these patients would be hospitalized and that hospitalization rates would vary between institutions.[5–7]

## Methods

NEDS is a stratified probability sample of approximately 20% of all hospital-based US EDs.[13] It includes ICD-9 codes, patient demographics, and hospital characteristics. Observations are made at the visit level, and are weighted according to the sampling scheme, which allows for national estimates. All data are anonymous and publicly available, and the study was considered exempt by our IRB.

For 2006–2014, we selected patients with Healthcare Cost and Utilization Project (HCUP) Clinical Classification Software (CCS) codes 11–45, indicating cancer. We then searched all diagnostic fields for codes indicating both fever (7806, 78060 and 7806), and neutropenia (2880, 28809, 28800, 28803, 28850, 2885 and 28859), in order to derive our sample of interest. We excluded visits ending in death or transfer to another facility. Visits with missing data were also excluded from the model.

Our main outcome is the proportion of FN ED visits ending in hospitalization. Our secondary outcomes are hospitalization rates by age, sex, median household income, payer, and hospital teaching status; and inter-hospital variation in FN hospitalization rates, calculated via the intra-class correlation coefficient (ICC). The ICC quantifies the proportion of total variation that is attributable to inter-hospital differences in hospitalization rate.

We fit a logistic regression model with hospitalization rate as the outcome, hospital as the exposure and the following covariates: age, sex, median household income, payer, hospital status, year of visit, and number of FN visits per hospital. Variables were selected *a priori*. We used backward elimination to remove predictors with p≥0.05 and chose the final model based on the lowest Akaike Information Criterion. To calculate the ICC, we fit a multilevel mixed effects model, and calculated the ICC from the covariance parameter estimates.

## Results

There were 9,064,754 records with a cancer CCS code, indicating 40,806,565 visits by patients with cancer nationwide (95%Confidence Interval [CI] 39,165,555–42,447,575). Of all US ED visits, 3.5% were made by patients with cancer (95%CI 3.4%-3.6%). Among the cancer visits, 79,428 had FN, representing 361,456 US cancer-associated FN visits (95%CI 336,568–386,343). After exclusion of deaths and transfers, 348,868 visits were available for analysis (95%CI 324,104–373,632). For our main outcome, 94% of visits ended in hospitalization (95%CI 93–94%) (Table 1).

**Table 1:** Characteristics of US ED febrile neutropenia visits, 2006–2014.

Characteristics	Proportion of this Subgroup Hospitalized % (95%CI)	Number of Patients	Percent
**Overall**	94 (93–94)	348,868	100
**Age (years)**			
≤ 3	91 (89–93)	13,260	4
4–9	89 (87–91)	19,092	5
10–14	92 (89–94)	9,444	3
15–19	93 (91–95)	8,226	2
20–29	95 (94–96)	11,917	3
30–39	93 (92–94)	16,056	5
40–49	93 (93–94)	32,171	9
50–59	94 (93–94)	59,680	17
60–69	94 (94–95)	82,574	24
70–79	95 (95–96)	68,558	20
80–89	96 (96–97)	26,069	7
≥ 90	97 (96–99)	1,821	1
**Sex**			
Male	94 (94–95)	172,223	49
Female	94 (93–94)	176,630	51
**Median household income quartile**			
1 (lowest)	93 (93–94)	72,320	21
2	93 (92–94)	85,581	25
3	94 (94–95)	88,855	25
4 (highest)	95 (94–95)	93,820	27
**Primary Health Insurance**			
Public	95 (95–95)	197,511	57
Private	92 (91–93)	133,919	38
Self-pay	92 (91–94)	6,309	2
Other	93 (92–95)	10,649	3
**Hospital Status**			
Metropolitan Teaching	95 (95–96)	207,960	60
Metropolitan, Non-teaching	94 (93–94)	110,530	32
Non-Metropolitan	84 (82–85)	30,378	9

Abbreviations: ED, emergency department; CI, confidence interval

Age, payer category, and hospital status were associated with likelihood of hospitalization (Table 2). Each decade of age conferred 1.23 times the odds of hospitalization. A patient presenting to a metropolitan teaching hospital was nearly seven times as likely to be hospitalized as a patient presenting to a non-metropolitan hospital (95% versus 84%, respectively). A given hospital’s number of FN ED visits per year did not predict the odds of hospitalization (odds ratio [OR] 1.00, 95%CI 1.00–1.00). Adjusting for age, primary payer, visit year, number of FN ED visits per hospital and hospital status, 26% of the variability in hospitalization rates was attributable to hospital-to-hospital variability.

**Table 2:** Multilevel mixed effects model for cancer associated febrile neutropenia hospitalization Rates, 2006–2014.

	OR (95%CI)	P—value
**Age (decades)**	1.23 (1.19–1.28)	<0.001
**Sex**		
Male	Reference	
Female	0.96 (0.89–1.03)	0.23
**Primary Health Insurance**		
Public	Reference	
Private	0.75 (0.69–0.82)	<0.001
Self-pay	0.74 (0.56–0.98)	0.03
Other	0.76 (0.58–1.002)	0.052
**Visit Year**		
2006–2008	Reference	
2009–2011	0.95 (0.81–1.11)	0.51
2012–2014	1.10 (0.93–1.30)	0.27
**Hospital Status**		
Metropolitan Teaching	Reference	
Metropolitan, Non-teaching	0.64 (0.54–0.76)	<0.001
Non-Metropolitan	0.15 (0.12–0.18)	<0.001
**ICC % (95%CI): Adjusted for Hospital Random Effects only**		34 (31–37)
**ICC % (95%CI): Adjusted for Hospital Random Effects & Patient and Hospital Characteristics**		26 (23–29)

Abbreviations: OR, odds ratio; CI, confidence interval; FN, febrile neutropenia; ICC, intra-class correlation coefficient

## Discussion

We found that 94% of all US FN ED visits end in hospitalization, confirming our *a priori* hypothesis, and consistent with our prior finding of a 95% hospitalization rate at one institution.[7] Hospitalization rates were slightly lower for pediatric patients, but still >90% for the entire cohort. Near-universal hospitalization of FN patients is problematic, because hospitalization and prolonged broad-spectrum antibiotic treatment carry risks including *C*. *difficile* colitis, selection of drug-resistant strains, drug toxicity, allergic reactions, drug-drug interactions, acquisition of nosocomial pathogens, exposure to medical errors, catheter-associated infections, thromboembolism, and financial burdens.[5,6]

Why are nearly all cancer-associated FN ED patients hospitalized? Guidelines recommend use of the MASCC score and other clinical predictors to determine which adult patients with cancer should be hospitalized.[5,6] Clinicians are asked to calculate the MASCC score, and for patients with a score >20, consider discharge if no fewer than 41 additional criteria are fulfilled (see Table 4 in [6]).[6] This risk stratification method is too cumbersome to be applied in real-life emergency medicine practice. Moreover, the MASCC score itself is insufficiently accurate, with a negative predictive value for complications of only 83%.[11] A newer score, the Clinical Index of Stable Febrile Neutropenia, applies only to solid tumor patients. In summary, current risk stratification tools are cumbersome and insufficiently accurate which likely explains why 94% of cancer-associated FN ED visits resulted in hospitalization.

We observed variation in hospitalization rates in metropolitan vs. non-metropolitan hospitals and across insurance categories. Of variability in hospitalization rates, 26% was explained by which hospital the patient visited. Cancer centers tend to be located in metropolitan areas, and it may be that patients with cancer who experience FN would visit EDs associated with cancer centers are more likely to be hospitalized than their counterparts visiting non-metropolitan EDs. This would be perplexing, however, since we would expect EDs affiliated with cancer centers to be more adept at individualizing care.

Our study has the following limitations. Case ascertainment depended on diagnostic codes, which may be subject to error. The codes we used to indicate neutropenia did not specify a neutrophil count of <500/μL^2^ or 1,000/μL^2^, both of which are accepted definitions.[5,6,14] However, we think emergency providers are unlikely to diagnose neutropenia in the absence of abnormalities of this magnitude, and the high rate of hospitalization we observed suggests that this was indeed a sample with bona fide immunosuppression.

The lack of a single discharge code describing FN may have also led to an underestimate of the true number of cases, and our findings of a subset of the combined discharge codes could be a biased sample towards sicker patients. In some patients, hospitalization is also based on non-clinical factors, such as psychosocial barriers to outpatient care (e.g., lack of transportation to clinic visits). However, such factors cannot explain our observation that nearly all of these patients were admitted. In addition, NEDS does not track revisits by the same patient, so some patients may be counted more than once. However, this does not detract from the importance of our finding that the vast majority of FN patients are admitted. NEDS also provides limited data on comorbidities that could influence the likelihood of hospitalization.

In conclusion, we have shown that nearly all cancer-associated FN ED visits end in hospitalization, that discharge is much more likely in non-metropolitan areas and for patients with insurance other than public insurance, and that 26% of variation in the hospitalization rate is due to which hospital the patient visited. We suggest that the available approaches for risk stratification may be too cumbersome and inaccurate to be useful, with the result being hospitalization of nearly all patients, including some who might safely be managed as outpatients. Simple, objective, valid risk stratification tools are needed.
